# Evaluation of WBSF, Color, Cooking Loss of *Longissimus Lumborum* Muscle with Fiber Optic Near-Infrared Spectroscopy (FT-NIR), Depending on Aging Time

**DOI:** 10.3390/molecules24040757

**Published:** 2019-02-20

**Authors:** Jarosław Wyrwisz, Małgorzata Moczkowska, Marcin Andrzej Kurek, Sabina Karp, Atanas G. Atanasov, Agnieszka Wierzbicka

**Affiliations:** 1 Division of Engineering in Nutrition, Department of Technique and Food Development, Warsaw University of Life Sciences (WULS-SGGW) 159c Nowoursynowska, 02-776 Warsaw, Poland; malgorzata_moczkowska@sggw.pl (M.M.); marcin_kurek@sggw.pl (M.A.K.); sabina_karp@sggw.pl (S.K.); agnieszka_wierzbicka@sggw.pl (A.W.); 2 Institute of Genetics and Animal Breeding of the Polish Academy of Sciences, 05-552 Jastrzebiec, Poland; atanas.atanasov@univie.ac.at; 3 Department of Pharmacognosy, University of Vienna, 1090 Vienna, Austria

**Keywords:** FT NIR spectroscopy, beef, WBSF, quality prediction

## Abstract

Near-infrared spectroscopy is a known technique for assessing the quality of compounds found in food products. However, it is still not widely used for predicting physical properties of meat using the online system. This study aims to assess the possibility of application of a NIR equipped with fiber optic system as an online measurement system to predict Warner–Bratzler shear force (WBSF) value, cooking loss (CL), and color of *longissimus lumborum* muscle, depending on aging time. The prediction model satisfactorily estimated the WBSF on day 1 and day 7 of aging as well as a* color parameter on day one and CL on day 21. This could be explained by the fact that during beef aging, the physicochemical structure of meat becomes more uniform and less differentiation of raw data is observed. There is still a challenge to obtain a verifiable model for the prediction of physical properties, using NIR, by utilizing more varied raw data.

## 1. Introduction

Consumers’ perceptions of meat quality, especially beef, have been a focus of much of previous works [1,2,3,4]. Beef quality attributes, which are important for consumers, can be divided into two groups: intrinsic and extrinsic [5]. The color, marbling, overall appearance, tenderness, juiciness, and palatability, as well as nutritional value and its change during the aging process, and other attributes that cannot be altered without altering the product’s nature, are intrinsic characteristics. Meanwhile, extrinsic attributes are features related to the product that are linked to “external” factors such as production and processing practices [2,6]. It is worth noting that the importance of individual quality indicators of beef has been changing over recent years as a result of increased consumer awareness [7,8], but tenderness remains a major attribute in determining overall meat quality; it is often considered as the crucial quality attribute of meat [9].

Beef tenderness changes during a muscle’s conversion into a meat product as a result of the proteolysis process. The post-slaughter protein degradation progress in meat is strongly related to aging process conditions. Therefore, monitoring of changes in beef tenderness during aging is necessary [10].

For this reason, the aging process for obtaining appropriate beef tenderness is required, which has been well-documented [10,11,12,13]. However, for how long this process should be carried out remains unknown. Beef tenderness also depends on the amount of intramuscular fat [14,15]. A high content of intramuscular fat results in more tender meat, because fat has a softer structure than muscle tissue. Additionally, the intramuscular fat content contributes to juiciness and improves palatability [16,17].

Despite the fact that beef tenderness is the primary consideration for consumer satisfaction, tenderness is not a factor directly incorporated into the quality grading process. To improve beef tenderness and overall quality, identification of tough carcasses must be a top priority for the beef industry. To preserve the current market and acquire new ones, the meat industry needs to produce consistent and high-quality beef. For this purpose, appropriate tools that are already available in the technological line are required to assess the quality of meat. There are many methods for measuring quality attributes (tenderness, color, flavor, water holding capacity (WHC), etc.) of culinary beef cuts, but only after leaving the meat slaughterhouse and under laboratory conditions. However, measurements to indicate the final quality of beef are not well developed yet. Time-consuming traditional methods are not appropriate for online assessment of different levels of beef quality. Therefore, a quick, accurate, and non-destructive method appropriate for online application [18,19] must be developed for modern processing plants. Near-infrared spectroscopy (NIRS) can offer this functionality due to the speed and ease of use and the ability to evaluate multiple quality parameters in one measurement. Near-infrared (NIR) technology provides complete information about the molecular bonds and chemical constituents in a scanned sample, so it is a convenient tool not only for characterizing foods, but also for quality measurements and process control [20]. Optical devices coupled to computers offer potentially high-speed data acquisition that may permit decision-making on meat quality, albeit from a selected small surface area only [21]. The meat industry has successfully used this technique for the prediction of chemical composition parameters such as fat, water, and protein content [22,23,24]. There are many publications related to the possibilities of using NIRS in testing the physical properties of meat [25,26]. On the basis of the NIR spectra of compounds found in the product, many researchers studied the prediction of meat tenderness [27,28]. However, few publications describe the use of near-infrared techniques to assess the quality of beef depending on aging time and their results are not clear. Therefore, this study aims to assess the potential of NIRS equipped with a fiber optic system (FOS) as an online measurement system to predict the Warner–Bratzler shear force (WBSF) value, cooking loss (CL), and color of *longissimus lumborum* (LL) muscle depending on aging time.

## 2. Results and Discussion

### 2.1. Characterization of Raw Material—Beef LL

Table 1 shows the descriptive data of pH and basic composition of LL muscle at 48 h post-mortem. The observed results indicate that the raw meat was of normal quality (without defects) according to Adzitey and Nurul [29]; thus, the obtained beef muscles were the subjects of the study.

With the extension of aging time, a decrease in water content in the meat is observed, which is correlated with the amount of drip loss. The amount of drip loss is due to the post-mortem proteolysis of cytoskeletal proteins [30,31]. Consequently, these processes have a significant impact on increasing the percentage share of other nutritional components, such as protein, fat, connective tissue, and ash.

### 2.2. Reference Data

The mentioned reference values of beef LL muscle WBSF, color, and CL according to the aging day are summarised in Table 2. WBSF, color, and CL analysis suggest that LL steaks were generally more tender (lower WBSF), slightly lighter (higher L*), redder (higher a*), more yellowish (higher b*), and with higher CL as aging time increased. The reduction of WBSF and the increment of CL are due to the degradation of proteins in the aging process [10]. The increased beef color values after aging are related to the influence limited by the enzymatic activity during vacuum aging, which resulted in a deeper O_2_Mb layer that is created in the presence of air oxygen, and blooming process occurs faster and more intensively [32]. Moreover, a higher lightness L*, after aging, can be explained by the protein degradation process during aging, leading to weakening of the protein structures, which results in higher light dispersion, thus increasing the lightness of the meat [10,33].

The highest coefficient of variation (CV) was reported for WBSF (>23%). However, the largest CV for WBSF was obtained for LL samples in D7 and D21 (27.8% and 27.54%, respectively). The lowest CV was L* lightness (<8%), irrespective of aging time. However, in the case of redness a*, yellowness b*, and CL muscle in each day of aging, CV ranges were 11.38–15.44, 12.44–14.76, and 13.02–16.41 respectively. The value of the CV indicates that the data can guarantee significant calibration [34]. According to Andrés, [35], the acceptable CV level should be no less than 20%, while De Marchi [25] claims that a range of CV 6%–19% for color index indicates the existence of exploitable variability in developing calibration models.

### 2.3. Chemometric Data Analysis

**Figure 1:**
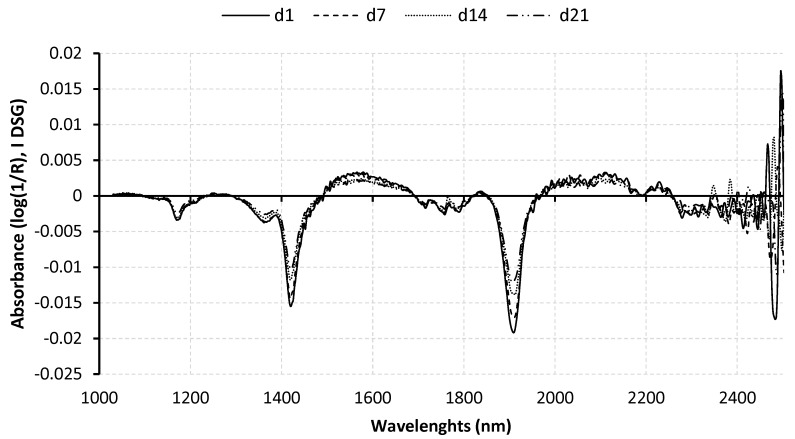
Average first derivatives Savitzky–Golay (I DGS) pre-treatment spectra (log(1/R)) of *longissimus lumborum* samples aged for 1 (d1), 7 (d7), 14 (d14) and 21 (d21) days.

The mean raw spectrum [log (1/R)], corresponding to the complete sample set for each aging time (89 LL samples), is represented in Figure 1. The first derivatives Savitzky–Golay (I DGS) pre-treatment spectra of the meat samples are dominated by the water absorbance because there are two broad peaks around 1460 and 1920 nm due to different forms of vibration of O–H bonds (1st and 2nd overtone, respectively). Additionally, the minor NIR absorption bands in fat are a CH_2_ second overtone around 1200 nm, CH_2_ first overtones at around 1730 and 1770 nm, and CN bonds in the range of 2050–2100 nm [36]. There are no remarkable absorption bands in the region of 2000–2500 nm, probably because these are obscured by the water signal [37].

In general, the average spectral signatures of 1d, 7d, 14d, and 21d samples had similar forms. However, they were only different in the magnitude of absorbances (log(1/R)). The average log(1/R) values of 1d aged samples were slightly higher than the 7d, 14d, and 21d aged samples throughout the spectral range. Similar spectral dependences of tender and tough samples were reported previously in predicting beef tenderness, using spectroscopy [38]. The meat aged 1d had a higher absorption rate than the meat aged 7d, 14d, and 21d at most wavelengths, particularly for the wavelengths between 1150 and 1300 nm and between 1600 and 1800 nm. These are the absorption bands in the fat regions, indicating changes in fat content. The changes in the fat content may result mainly from higher drip loss during longer meat aging as shown in Table 1.

### 2.4. Prediction of Instrumental WBSF, Color, and CL

Many different methods for beef tenderness prediction have been developed, such as computer vision [39], hyperspectral imaging [40], and also spectroscopy in near-infrared (NIR) regions of the electromagnetic spectrum [41]. Table 3 summarizes the results of prediction models for WBSF, color (L*, a*, b*), and CL of LL muscle aged D1, D7, D14, and D21 in calibration and validation sets based on spectra after using different pre-treatments: SNV, MSC, I DSG or II DSG. Preliminary analysis indicated that the calibration models without any pre-treatment had a higher number of latent variables (L_v_) and lower coefficient of determination R^2^_c_.

Partial least squares (PLS) models to predict WBSF were optimal for samples after each aging day. WBSF models’ prediction had the highest R^2^_c_ (0.71), and R^2^_p_ (0.67) at the lowest standard error of calibration (SEC) (4.81 N) and standard error of prediction (SEP) (4.85 N) were obtained for samples aged 7d, using I DSG spectral pre-treatment routine, which resulted in a residual prediction deviation (RPD) of 2.18. Bias was negative and close to zero (0.15), indicating that the predictive model slightly overestimated WBSF values. The larger RPD of WBSF (2.48) was obtained for 1d LL samples, although R^2^_c_ and R^2^_p_ were smaller (0.65 and 0.62, respectively), which was due to the larger SD and slightly lower SEP than 7d aged samples. The predictive model underestimated WBSF values slightly (Bias = 0.15). A similar tendency was noted by Liu et al. (26). However, results obtained in this study were higher compared to results reported by Cluff et al. [42], who obtained the correlation coefficient for calibration and validation at about 0.67 for striploin, but lower than those reported by Saadatian et al. [40] for different beef muscles (top blade, tenderloin, rib eye, and top sirloin). The higher value of R^2^_c_, reported by Saadatian et al. [40], was probably related to higher wavelengths in NIR (900–1700 nm) compared to NIR spectra of 400–1000 nm, which could have been more effective in sensing chemical constituents such as protein, water, and fat. Also, the differences in correlation coefficient value may result from the use of different muscles.

Of the color coordinates, the best prediction results were characterized by redness. Moreover, only the model for samples aged 1d had RPD > 2 (2.15), with moderate values of R^2^_c_ (0.60) and R^2^_p_ (0.57). This model was also the best when using the PLS and I DSG spectral pretreatment regression and caused a slight underestimation of a* values (bias = 0.14). When the MSC pretreatment was used for the PCR models to predict the L*, the R^2^_c_, R^2^_p,_ and RPD were highest for the LL samples aged 7d, although RPD was less than two, which, according to Prieto [43], indicates the low predictive utility of this model. The low RPD is because of the variability of the results for samples on day one of aging, which was higher than the variability of results in the later days of aging. This is due to the chemical changes, occurring over time. This corresponds, for example, with the decreasing water content as a function of time, and thus chemical changes take place more slowly [44]. However, Liu et al. [26] received quite high R^2^ for predicting a* and b* values and was relatively low for the model predicting the L* value, which are the opposite results of our study. Adjustment of the meat color prediction model is closely related to myoglobin content and its form due to the different molecular environment of heme vibrations. Low prediction coefficients may be due to the small variability of the basic LL muscle composition, and thus the obtained spectra, which in effect reduced the range of calibration and effectiveness of prediction.

In the case of CL, the highest R^2^_c_ and R^2^_p_ values (0.72 and 0.66, respectively) and RPD (2.05), with limiting SEC (2.54), SEP (1.89), L_v_ (4) and bias (−0.11) by using LL samples aged 21d, were observed. These results are similar to those found by De Marchi [25] and are higher compared to Prieto et al. [45].

## 3. Materials and Methods

### 3.1. Raw Material and Preparation

The muscle *longissimus lumborum (LL)* was extracted at a local slaughterhouse from the left and right sides of 89 beef carcasses from Holstein Friesian, with 62 bulls (average cold carcass weight = 323 kg ± 16 kg) and 27 heifers (average cold carcass weight = 245 kg ± 11 kg), which were 16–26 months of age. The animals were slaughtered in accordance with internal regulations and under Polish General Veterinary Inspectorate inspection. Carcasses were classified using the SEUROP classification scale for conformation (S—superior; E—excellent; U—very good; R—good; O—fair; P—poor). The fat cover classification consists of a 1–5 scale: 1—low, 2—slight, 3—average, 4—high, 5—very high. Cold carcasses were graded as R to O-, conformation with fat cover of 1+ to 3+. For each carcass, the LL muscle was removed (freed of external fat and visible connective tissue) 48 h postmortem, chilled to 3 °C and cut into 5–6 steaks 2.5 cm thick from each LL muscle (left and right, respectively), with a sample weight of 240 g ± 22 g. These were randomly assigned (for left and right side of carcass seperately) to the next stages of experiments. Each steak (N = 178 per individual aging time) was placed individually in PA/PE bags and vacuum packaged, using a Vac-20 SL2A packaging machine (Edesa Hostelera S.A., Barcelona, Spain). The steaks were then wet-aged at 2 ± 1 °C for 1 d, 7 d, 14 d, and 21 d. At each aging time, steaks were removed from the vacuum package and were bloomed while protecting from light for 30 min at 2 ± 1 °C on plastic trays, and NIR scanning and instrumental color measurement were done. The steaks were then re-packaged with vacuum and blast frozen (−25 °C) for subsequent cooking for WBSF and CL.

### 3.2. NIR Spectroscopy Technology

Whole meat samples were scanned after 30 min of bloom, using a NIRFlex N-500 FT-NIR spectrometer with Fiber-Optic Solids (FOS) probe equipped with the software NIRWare (Büchi Labortechnik AG, Flawil, Switzerland). A FOS probe (2 m length, 4 mm outer diameter optical fibers, and 560 optical fibers in bundle) was used to transmit reflected light from the sample surface to extended range InGaAs detector (temperature controlled) and polarization interferometer with TeO_2_ wedges. A 20-W tungsten halogen was used as the light source. Each steak was placed on a flat surface and scanned at the top of the muscle in six different locations by holding the probe on the muscle fiber in a perpendicular orientation (n = 6). Thirty-two scans were averaged for every spectrum for one location. These six measurements/spectrum were then averaged to obtain the spectral data for the meat samples. Samples were scanned in reflectance mode from 10,000 to 4000 cm^−1^ at 8 cm^−1^ resolution. A ceramic tile or internal reference panel was used as a white reference between each sample.

### 3.3. Spectrometric Quantification of Basic Meat Composition

Beef composition (water, fat, protein, and total connective tissue content) was determined using a near-infrared spectrometer NIRFlex N-500 (Büchi) (Flawil, Switzerland). Measurements were taken using a NIRFlex Solids module of spectral range 12,500–4000 cm^−1^ in reflectant mode. Meat portions of 100 g were homogenized and placed in a petri dish, covering the surface with a 0.5 cm layer. Three measurements of each sample (n = 3) were taken at a 32 scanning rate (23).

### 3.4. pH Value Evaluation

The pH value of muscles was measured in LL muscle according to the PN-ISO 917:2001/Ap1:2002 standard. pH-metric results were obtained by using a Testo 205 series pH-meter (Pruszków, Poland) equipped with an insertion glass electrode, which was placed directly into the samples (2 cm deep into the steaks). Each measurement was performed in three repetitions (n = 3), taking the mean value as the result. The temperature of the samples during measurements was 2 ± 1 °C.

### 3.5. Instrumental Color Measurement in CIE L* a* b* System

Instrumental color analysis of beef was determined, using a Minolta CR-400 chromometer calibrated against a white plate (L* = 98.45, a* = −0.10, b* = −0.13), using an 8 mm aperture, illuminate D65 (6500 K color temperature) at a standard observation of 2°. L* (lightness ranged from 0 to 100), a* (color axis ranged from greenness (−a*) to redness (+a*)), and b* (color axis ranged from blueness (−b*) to yellowness (+b*)). Color measurements of the steak were taken from five locations, including every quarter and the centers of the surfaces, and the mean value was taken by taking the average of five measurements (n = 5) [46]. Data were collected within a 30 min blooming period under refrigerated conditions (2 ± 1 °C).

### 3.6. Warner–Bratzler Shear Force Determination

After aging, meat samples were prepared for shear force analysis according to Wyrwisz et al. [32]. The steaks (100 ± 10 g) were cooked individually in closed PA/PE bags immersed in a water bath (Memmert, WNE 14, Schwabach, Germany) at 80 °C, to achieve a final internal temperature of 71 °C. They were subsequently cooled down in cold water and stored overnight at 3 ± 1 °C. Instrumental measurement of WBSF was conducted using a universal testing machine, Instron (Model 5965, Norwood, MA, USA), with a Warner–Bratzler shear attachment, consisting of a V-notch blade, according to Wyrwisz et al. [29]. Six cores (1.27 cm in diameter and 2.5 ± 0.2 cm in length) were obtained from each steak, parallel to the muscle fiber’s orientation. A 500 N load cell was used, and the crosshead speed was set at 200 mm/min.

### 3.7. Cooking Loss

The percentage of CL was determined through measurement of sample mass before (M_i_) and after heat treatment and after cooling to ambient temperature (M_f_) (n = 3). Heat treatment was performed as in Section 3.6. CL was calculated according to the Equation (1)

(1) $$\mathrm{CL}=\left(1-\frac{M_{f}}{M_{i}}\right)\cdot 100\%,$$

### 3.8. Statistical Analysis Data Analysis

The chemometric software NIRCal 5.5.3000 (Büchi Labortechnik AG, Flawil, Switzerland) was used to create the quantitative calibration and validation models. Two thirds of the spectra was used as calibration set and 1/3 as validation set. The spectral data points obtained from the spectrometers were processed, using multivariate data analysis with the purpose of developing calibration models for predicting the reference information from the WBSF, L*, a*, b*, and CL. Predictions based on spectral information were performed with partial least squares regression PLSR (after centering of the data) or principal component regression (PCR) with optimal number latent variables (Lv) by describing the maximum covariance between the spectral information and references. Spectral pre-treatments data, such as standard normal variate (SNV), multiplicative scatter correction (MSC) and first or second derivatives Savitzky-Golay (I DSG and II DSG, respectively), were applied to the spectra with the purpose of reducing noise and scattering effects and, thus, to obtain the optimal calibration model [47].

In the internal full cross-validation process, the prediction capability of the model was evaluated, using the coefficient of determination in calibration (R^2^_c_), standard error of calibration (SEC), coefficient of determination in prediction (R^2^_p_), and standard error of prediction (SEP). Moreover, the prediction models were evaluated using the residual prediction deviation (RPD = SD/SEP) [34]. An RPD value above 3 is considered adequate for routine analysis, between 2 to 3 represents a good prediction, and less than 1.5 indicates incorrect prediction and the model cannot be used for prediction [43]. The best model was selected with respect to the highest R^2^_p_ and RPD, the lowest SEP, and limitation of the number of latent variables (<10) [43]. Moreover, the accuracy of the correlation (bias), calculated as the systematic difference between the predicted values (y_i_) and the measured values (x_i_), was determined (Equation (2)). The value of the bias should be close to zero for accurate calibration models.

(2) $$\mathrm{Bias}=\sum_{i=1}^{n}\left(x_{i}-y_{i}\right),$$

## 4. Conclusions

This study was conducted to assess the possibility of using Fourier transform near-infrared spectroscopy (FT-NIR) with a fiber optic probe to predict WBSF value, CL, and color of *longissimus lumborum* muscle, depending on aging time. To accomplish this, partial least squares (PLS) regression at optimal wavelengths was conducted. The results showed that FT-NIR has potential as an online and non-destructive tool for beef tenderness prediction. However, there is still a need to improve the predictive model’s accuracy. This could be rationalized by the fact that only on some days of aging, the values of RPD were above 2.

Even though the PLS model is not fully satisfactory for it to be implemented in the beef industry to predict tenderness, redness, and CL, improvement of this model could be applied for measuring WBSF, a*, and CL with high accuracy using non-destructive NIR measurement regardless of the aging day. Thanks to this, multi-parametric, non-destructive, and quick assessment of beef quality will be possible, indicating the minimum aging time determining the proper aging degree of beef, greater meat tenderness and more attractive color.

## Figures and Tables

**Table 1:** Mean ± standard deviation of pH and the basic meat composition of beef *longissimus lumborum* (LL) muscle (N = 178 for each aging time).

	Aging Time			
	D1	D7	D14	D21
pH	5.51 ± 0.15	5.55 ± 0.09	5.64 ± 0.11	5.68 ± 0.06
Water, %	74.51 ± 1.08	74.01 ± 1.05	73.11 ± 0.88	73.15 ± 0.79
Fat, %	2.44 ± 0.66	2.51 ± 0.46	2.58 ± 0.32	2.57 ± 0.48
Protein, %	22.41 ± 0.47	22.65 ± 0.87	23.24 ± 0.64	23.35 ± 1.01
Total connective tissue, %	1.47 ± 0.27	1.51 ± 0.38	1.62 ± 0.41	1.64 ± 0.35

**Table 2:** Reference values of Warner–Bratzler shear force (WBSF), color (L*, a*, b*), and cooking loss (CL) of beef LL muscle measured by traditional methods (N = 178 for each aging day).

	Aging Day	Mean	SD	Range		CV, %
				min	max	
WBSF [N]	D1	44.60	11.97	22.01	77.64	26.85
	D7	38.04	10.57	20.65	66.73	27.80
	D14	33.27	7.78	15.69	59.81	23.37
	D21	30.95	8.53	13.06	57.98	27.54
L* [%]	D1	37.49	2.75	30.43	45.19	7.33
	D7	38.97	2.99	31.89	46.67	7.67
	D14	39.97	3.14	32.06	47.37	7.86
	D21	39.50	2.77	32.85	45.89	7.02
a*	D1	18.97	2.52	13.36	25.13	13.28
	D7	21.49	2.45	15.47	26.21	11.38
	D14	21.98	2.65	15.87	28.21	12.06
	D21	21.72	3.35	16.11	26.01	15.44
b*	D1	8.92	1.18	5.06	11.74	13.29
	D7	10.07	1.46	5.61	13.00	14.54
	D14	10.39	1.53	5.45	13.38	14.76
	D21	9.57	1.19	5.57	12.72	12.44
CL [%]	D1	28.34	3.69	21.74	37.22	13.02
	D7	29.22	3.85	22.91	35.54	13.18
	D14	31.13	4.52	21.82	36.23	14.52
	D21	31.75	5.21	20.33	35.11	16.41

SD—standard deviation; CV—coefficient of variation; D1, D7, D14, D21—LL samples aged 1, 7, 14, 21 days, respectively.

**Table 3:** NIRS calibration and prediction statistics for WBSF, color (L*, a*, b*) and CL of LL muscle.

		Spectral Pretreatment	Model	No. of L_v_	R^2^_c_	SEC	R^2^_p_	SEP	RPD	Bias
WBSF [N]	D1	SNV	PLS	3	0.65	4.22	0.62	4.83	2.48	0.15
	D7	I DSG	PLS	5	0.71	4.81	0.67	4.85	2.18	−0.12
	D14	SNV	PLS	5	0.54	4.89	0.49	5.02	1.55	−0.72
	D21	SNV	PLS	3	0.22	5.12	0.31	6.58	1.30	−0.96
L* [%]	D1	MSC	PLS	3	0.46	2.4	0.33	2.79	0.98	−1.06
	D7	MSC	PCR	7	0.84	1.87	0.87	1.52	1.97	−0.27
	D14	SNV	PCR	5	0.34	2.91	0.35	2.89	1.09	0.88
	D21	MSC	PLS	8	0.12	1.74	0.23	1.85	1.50	0.43
a*	D1	I DSG	PLS	6	0.60	1.08	0.57	1.17	2.15	0.14
	D7	I DSG	PLS	4	0.22	2.18	0.35	2.01	1.22	−0.75
	D14	I DSG	PLS	6	0.15	2.63	0.21	2.61	1.02	−0.83
	D21	SNV	PLS	5	0.21	4.45	0.46	2.16	1.55	−0.97
b*	D1	I DSG	PLS	4	0.61	0.95	0.61	0.97	1.22	0.72
	D7	MSC	PCR	3	0.44	1.25	0.42	1.26	1.16	−0.95
	D14	I DSG	PCR	3	0.34	1.44	0.34	1.12	1.37	1.01
	D21	II DSG	PLS	2	0.25	0.98	0.29	1.11	1.07	0.75
CL	D1	I DSG	PLS	3	0.64	3.49	0.47	3.64	1.01	0.83
	D7	MSC	PCR	5	0.13	6.03	0.07	6.12	0.63	1.23
	D14	SNV	PLS	4	0.23	3.94	0.27	3.9	1.16	−0.87
	D21	SNV	PLS	4	0.72	2.54	0.66	1.89	2.05	−0.11

D1, D7, D14, D21—LL samples aged 1, 7, 14, 21 days, respectively; SVN—standard normal variate; MSC—multiplicative scatter correction; I DSG, II DSD first or second derivatives Savitzky–Golay, respectively; PLS—partial least squares regression; PCR—principal component regression.

## References

[1] Banović M., Grunert K.G., Barreira M.M., Fontes M.A. (2009). Beef quality perception at the point of purchase: A study from Portugal. Food Qual. Preference.

[2] Henchion M.M., McCarthy M., Resconi V.C. (2017). Beef quality attributes: A systematic review of consumer perspectives. Meat Sci..

[3] Henchion M., McCarthy M., Resconi V.C., Troy D. (2014). Meat consumption: Trends and quality matters. Meat Sci..

[4] Troy D.J., Kerry J.P. (2010). Consumer perception and the role of science in the meat industry. Meat Sci..

[5] Hocquette J.F., Botreau R., Picard B., Jacquet A., Pethick D.W., Scollan N.D. (2012). Opportunities for predicting and manipulating beef quality. Meat Sci..

[6] Żakowska-Biemans S., Pieniak Z., Gutkowska K., Wierzbicki J., Cieszyńska K., Sajdakowska M., Kosicka-Gębska M. (2016). Beef consumer segment profiles based on information source usage in Poland. Meat Sci..

[7] Uys P., Bisschoff C. (2016). Identifying consumer buying preferences of beef. Probl. Perspect. Manag..

[8] Verbeke W., Pérez-Cueto F.J.A., de Barcellos M.D., Krystallis A., Grunert K.G. (2010). European citizen and consumer attitudes and preferences regarding beef and pork. Meat Sci..

[9] Cassens A.M., Arnold A.N., Miller R.K., Gehring K.B., Savell J.W. (2018). Impact of elevated aging temperatures on retail display, tenderness, and consumer acceptability of beef. Meat Sci..

[10] Moczkowska M., Półtorak A., Wierzbicka A. (2017). The effect of ageing on changes in myofibrillar protein in selected muscles in relation to the tenderness of meat obtained from cross-breed heifers. Int. J. Food Sci. Technol..

[11] Colle M.J., Richard R.P., Killinger K.M., Bohlscheid J.C., Gray A.R., Loucks W.I., Day R.N., Cochran A.S., Nasados J.A., Doumit M.E. (2015). Influence of extended aging on beef quality characteristics and sensory perception of steaks from the gluteus medius and longissimus lumborum. Meat Sci..

[12] Colle M.J., Richard R.P., Killinger K.M., Bohlscheid J.C., Gray A.R., Loucks W.I., Day R.N., Cochran A.S., Nasados J.A., Doumit M.E. (2016). Influence of extended aging on beef quality characteristics and sensory perception of steaks from the biceps femoris and semimembranosus. Meat Sci..

[13] Da Silva Coutinho M.A., Ramos P.M., da Luz e Silva S., Martello L.S., Pereira A.S.C., Delgado E.F. (2017). Divergent temperaments are associated with beef tenderness and the inhibitory activity of calpastatin. Meat Sci..

[14] Koohmaraie M., Geesink G.H. (2006). Contribution of postmortem muscle biochemistry to the delivery of consistent meat quality with particular focus on the calpain system. Meat Sci..

[15] Paredi G., Raboni S., Bendixen E., de Almeida A.M., Mozzarelli A. (2012). “Muscle to meat” molecular events and technological transformations: The proteomics insight. J. Proteom..

[16] Chriki S., Renand G., Picard B., Micol D., Journaux L., Hocquette J.F. (2013). Meta-analysis of the relationships between beef tenderness and muscle characteristics. Lives Sci..

[17] Lee S.H., Joo S.T., Ryu Y.C. (2010). Skeletal muscle fiber type and myofibrillar proteins in relation to meat quality. Meat Sci..

[18] Kucha C.T., Liu L., Ngadi M.O. (2018). Non-destructive spectroscopic techniques and multivariate analysis for assessment of fat quality in pork and pork products: A review. Sensors.

[19] Mileusnić I.M., Rosic J.Z.S., Munćan J.S., Dogramadzi S.B., Matija L.R. (2017). Computer assisted rapid nondestructive method for evaluation of meat freshness. FME Trans..

[20] Osborne B.G. (2000). Near-Infrared Spectroscopy in Food Analysis. Encycl. Anal. Chem..

[21] Vote D.J., Belk K.E., Tatum J.D., Scanga J.A., Smith G.C. (2003). Online prediction of beef tenderness using a computer vision system equipped with a BeefCam module. J. Anim Sci..

[22] Clarke R. (2014). On-Line measurement of meat quality. Encyclopedia of Meat Science.

[23] Wyrwisz J., Półtorak A., Zalewska M., Zaremba R., Wierzbicka A. (2012). Analysis of relationship between basic composition, pH, and physical properties of selected bovine muscles. Bull. Vet. Inst. Pulawy.

[24] Wyrwisz J., Półtorak A., Poławska E., Pierzchała M., Jóźwik A., Zalewska M., Wierzbicka A. (2012). The impact of heat treatment methods on the physical properties and cooking yield of selected muscles from Limousine breed cattle. Anim. Sci. Pap. Rep..

[25] De Marchi M. (2013). On-line prediction of beef quality traits using near infrared spectroscopy. Meat Sci..

[26] Liu Y., Lyon B.G., Windham W.R., Realini C.E., Pringle T.D.D., Duckett S. (2003). Prediction of color, texture, and sensory characteristics of beef steaks by visible and near infrared reflectance spectroscopy. A feasibility study. Meat Sci..

[27] Price D.M., Hilton G.G., VanOverbeke D.L., Morgan J.B. (2008). Using the near-infrared system to sort various beef middle and end muscle cuts into tenderness categories. J. Anim. Sci..

[28] Rust S.R., Price D.M., Subbiah J., Kranzler G., Hilton G.G., Vanoverbeke D.L., Morgan J.B. (2008). Predicting beef tenderness using near-infrared spectroscopy. J. Anim. Sci..

[29] Adzitey F., Nurul H. (2011). Pale soft exudative (PSE) and dark firm dry (DFD) meats: Causes and measures to reduce these incidences-a mini review. Int. Food Res. J..

[30] Kim J.-H., Kim D.-H., Ji D.-S., Lee H.-J., Yoon D.-K., Lee C.-H. (2017). Effect of aging process and time on physicochemical and sensory evaluation of raw beef top round and shank muscles using an electronic tongue. Korean J. Food Sci. Anim..

[31] Zeng Z., Li C., Ertbjerg P. (2017). Relationship between proteolysis and water-holding of myofibrils. Maet Sci..

[32] Wyrwisz J., Moczkowska M., Kurek M., Stelmasiak A., Półtorak A., Wierzbicka A. (2016). Influence of 21 days of vacuum-aging on color, bloom development, and WBSF of beef semimembranosus. Meat Sci..

[33] MacDougall D.B., Taylor A.A. (1975). Colour retention in freshly cut meat stored in oxygen a commercial scale trial. J. Food Techol..

[34] Furtado E.J.G., Bridi A.M., Barbin D.F., Barata C.C.P., Peres L.M., Da Costa Barbon A.P.A., Andreo N., De Lima Giangareli B., Terto D.K., Batista J.P. (2018). Prediction of pH and color in pork meat using VIS-NIR Near-infrared Spectroscopy. Food Sci. Technol..

[35] Andrés S., Giráldez F.J., López S., Mantecón Á.R., Calleja A. (2005). Nutritive evaluation of herbage from permanent meadows by near-infrared reflectance spectroscopy: 1. Prediction of chemical composition and in vitro digestibility. J. Sci. Food Agric..

[36] Hoving-Bolink A.H., Vedder H.W., Merks J.W.M., de Klein W.J.H., Reimert H.G.M., Frankhuizen R., van den Broek W.H.A.M., Lambooij E. (2005). Perspective of NIRS measurements early post mortem for prediction of pork quality. Meat Sci..

[37] Brøndum J., Munck L., Henckel P., Karlsson A., Tornberg E., Engelsen S.B. (2000). Prediction of water-holding capacity and composition of porcine meat by comparative spectroscopy. Meat Sci..

[38] Bowling M.B., Vote D.J., Belk K.E., Scanga J.A., Tatum J.D., Smith G.C. (2009). Using reflectance spectroscopy to predict beef tenderness. Meat Sci..

[39] Tian Y.Q., McCall D.G., Dripps W., Yu Q., Gong P. (2005). Using computer vision technology to evaluate the meat tenderness of grazing beef. Food Aust..

[40] Saadatian F., Liu L., Ngadi M.O. (2015). Hyperspectral imaging for beef tenderness assessment. Int. J. Food Process. Technol..

[41] Dixit Y., Casado-Gavalda M.P., Cama-Moncunill R., Cama-Moncunill X., Markiewicz-Keszycka M., Cullen P.J., Sullivan C. (2017). Developments and challenges in online NIR spectroscopy for meat processing. Compr. Rev. Food Sci. Food Saf..

[42] Cluff K., Naganathan G.K., Subbiah J., Lu R., Calkins C.R., Samal A. (2008). Optical scattering in beef steak to predict tenderness using hyperspectral imaging in the VIS-NIR region. Sens. Instrum. Food Qual..

[43] Prieto N., Roehe R., Lavín P., Batten G., Andrés S. (2009). Application of near infrared reflectance spectroscopy to predict meat and meat products quality: A review. Meat Sci..

[44] Watson A.D., Gunning Y., Rigby N.M., Philo M., Kemsley E.K. (2015). Meat authentication via multiple reaction monitoring mass spectrometry of myoglobin peptides. Anal. Chem..

[45] Prieto N., Andrés S., Giráldez F.J., Mantecón A.R., Lavín P. (2008). Ability of near infrared reflectance spectroscopy (NIRS) to estimate physical parameters of adult steers (oxen) and young cattle meat samples. Meat Sci..

[46] Półtorak A., Wyrwisz J., Moczkowska M., Marcinkowska-Lesiak M., Stelmasiak A., Rafalska U., Wierzbicka A., Sun D.W. (2015). Microwave vs. convection heating of bovine Gluteus Medius muscle: Impact on selected physical properties of final product and cooking yield. Int. J. Food Sci. Technol..

[47] Kapper C., Klont R.E., Verdonk J.M.A.J., Williams P.C., Urlings H.A.P. (2012). Prediction of pork quality with near infrared spectroscopy (NIRS) 2. Feasibility and robustness of NIRS measurements under production plant conditions. Meat Sci..

